# Understanding sharps injuries in home healthcare: The Safe Home Care qualitative methods study to identify pathways for injury prevention

**DOI:** 10.1186/s12889-015-1673-x

**Published:** 2015-04-11

**Authors:** Pia Markkanen, Catherine Galligan, Angela Laramie, June Fisher, Susan Sama, Margaret Quinn

**Affiliations:** Department of Work Environment, College of Health Sciences, University of Massachusetts Lowell, Lowell, (MA) USA; Occupational Health Surveillance Program, Massachusetts Department of Public Health, Boston, (MA) USA; Training for Development of Innovative Control Technology Project, San Francisco, (CA) USA

**Keywords:** Home healthcare, Bloodborne pathogens, Sharps injury prevention, Qualitative methods

## Abstract

**Background:**

Home healthcare is one of the fastest growing sectors in the United States. Percutaneous injuries from sharp medical devices (sharps) are a source of bloodborne pathogen infections among home healthcare workers and community members. Sharps use and disposal practices in the home are highly variable and there is no comprehensive analysis of the system of sharps procurement, use and disposal in home healthcare. This gap is a barrier to effective public health interventions. The objectives of this study were to i) identify the full range of pathways by which sharps enter and exit the home, stakeholders involved, and barriers for using sharps with injury prevention features; and ii) assess the leverage points for preventive interventions.

**Methods:**

This study employed qualitative research methods to develop two systems maps of the use of sharps and prevention of sharps injuries in home healthcare. Twenty-six in-depth interview sessions were conducted including home healthcare agency clinicians, public health practitioners, sharps device manufacturers, injury prevention advocates, pharmacists and others. Interview transcripts were audio-recorded and analyzed thematically using NVIVO qualitative research analysis software. Analysis of supporting archival material also was conducted. All findings guided development of the two maps.

**Results:**

Sharps enter the home via multiple complex pathways involving home healthcare providers and home users. The providers reported using sharps with injury prevention features. However, home users’ sharps seldom had injury prevention features and sharps were commonly re-used for convenience and cost-savings. Improperly discarded sharps present hazards to caregivers, waste handlers, and community members. The most effective intervention potential exists at the beginning of the sharps systems maps where interventions can eliminate or minimize sharps injuries, in particular with needleless treatment methods and sharps with injury prevention features. Manufacturers and insurance providers can improve safety with more affordable and accessible sharps with injury prevention features for home users. Sharps disposal campaigns, free-of-charge disposal containers, and convenient disposal options remain essential.

**Conclusions:**

Sharps injuries are preventable through public health actions that promote needleless treatment methods, sharps with injury prevention features, and safe disposal practices. Communication about hazards regarding sharps is needed for all home healthcare stakeholders.

## Background

Home healthcare (HHC) services are a growing industry in the United States. The U.S. Bureau of Labor Statistics projects “personal care aide” and “home care aide” to be the 2^nd^ and 3^rd^ fastest growing jobs during 2012–2022 [1]. In 2009, annual HHC expenditures were estimated at $72.2 billion [2], reflecting shorter hospital stays and more medical technologies adapted for home settings [1,3]. Sharp medical devices (collectively called “sharps”) including syringes with needles, infusion systems, lancets, and blood collection devices have become common in HHC. As a result, workers face a risk of sharps injuries and bloodborne pathogen (BBP) exposures, of which hepatitis B, hepatitis C, and human immunodeficiency virus (HIV) are the most concerning. Due to advancement in medical treatment and technologies, people are living longer with chronic conditions like HIV and hepatitis. Americans with HIV receive care more frequently in the home setting than in other health care settings [4].

A previous study by the research team found that approximately 35% of nurses and 6% of aides experienced at least one injury with a previously used sharp during their HHC career [5]. While caution needs to be exercised when comparing studies due to differences in data collection and analyses, the annual sharps injury incidence rate for home care nurses (5.1 per 100 full-time equivalent (FTE) nurses) [5] was consistent with the findings in hospitals and non-hospital facilities [6-9]. Although home care agencies routinely train staff on hazards associated with BBP exposures and exposure reporting protocols, difficulties exist in minimizing BBP hazards. Homes are more variable and less controlled than facility-based settings [10,11] and educating patients and families requires considerable resources and expertise. When sharps injuries occur, a medical facility to provide post-exposure care is not always nearby [11]. HHC clinicians are covered by the Occupational Safety and Health Administration’s (OSHA) Bloodborne Pathogens Standard which requires the use of engineering and work practice controls to eliminate or minimize BBP exposures among employees [12]. Engineering controls include sharps with injury prevention features (SIPFs), however, the previous study found that SIPFs were not frequently used in home care [5].

The previous study also estimated a sharps injury incidence rate for home care aides of 1.0 per 100 FTEs [5]. Although this might appear low, with the substantial number of aides in HHC, it translates to a large number of injuries. Most aides’ injuries are associated with improperly disposed sharps encountered during cleaning tasks [5]. Estimates suggest that about 8–9 million Americans use sharps to manage their health conditions at home, translating to more than 3 billion used sharps to be disposed of outside facility-based health care settings annually [13]. In the United States, aides employed by HHC agencies are supervised by a nurse and are not assigned medical procedures or tasks requiring sharps use. However, focus group findings suggest that aides are sometimes asked by a patient or family to assist with medical procedures, such as using a lancet or injecting medication – particularly if aides are hired directly by the patient [10]. A study led by the University of Maryland (USA) indicated that over a third of personal care assistants who reported patient blood contact were using a lancet or needle [14].

### Objectives

The main objectives of this study were to: i) identify the full range of pathways by which sharps enter and exit the home, stakeholders involved, practices for use and disposal, and barriers for using sharps with injury prevention features; and ii) assess the leverage points for preventive interventions. The study also summarized the analyses for concise public health communication.

## Methods

### Study population and recruitment

A total of 26 in-depth interview sessions were conducted between June 2011 and April 2013. The study employed both purposive and snowball participant recruitment sampling strategies through contacts with research partners and other stakeholders. Two sessions included two interviewees, thus, the total number of subjects interviewed was 28 (see Table 1). Nine sessions were conducted by phone and the rest in-person.

**Table 1:** **Safe Home Care sharps study: interview sessions conducted during June 2011–April 2013**

**Interview session #**	**Position/area of expertise**	**Organization**
1.	Infection preventionist	Private HHC agency A in Massachusetts (MA)
2.	Safety and health officer	Labor Union in MA
3.	Safety and health officer	Labor Union in MA
4.	Education program coordinator	Private HHC agency B in MA
5.	Hospice clinical services coordinator	Private HHC agency A in MA
6.	IV therapy/clinical services coordinator	Private HHC agency A in MA
7.	Education program coordinator	Private HHC agency C in MA
8.	Clinical coordinator	Private HHC agency C in MA
9.	Pharmacist	Pharmacy owner in a MA town/city
10.	Physician, community outreach and prevention specialist	Non-government organization (NGO) in MA
11.	Diabetes awareness/ environmental health and safety services (2-person interview)	NGO on environmental services outside MA
12.	Sharps injury prevention specialist	Independent specialist outside MA
13.	Executive director	Sharps manufacturer A outside MA
14.	Chief executive officer	Sharps manufacturer B outside MA
15.	Clinical manager	Sharps manufacturer C outside MA
16.	Clinical specialist	Sharps manufacturer C outside MA
17.	Diabetes care manager/diabetes educator	Healthcare organization in MA
18.	Physician and founder of an NGO for sharps injury prevention	Independent specialist outside MA
19.	Diabetes educator	Healthcare organization in MA
20.	Founder of an NGO for sharps injury prevention	Independent specialist outside MA
21.	Director	Sharps manufacturer D outside MA
22.	Primary care physician	Healthcare organization in MA
23.	Health agent	MA town/city
24.	Occupational and environmental health consultant	Independent specialist outside MA
25.	Pharmacist/ academic pharmacy researcher	University outside MA
26.	Public health nurse manager & public health director (2-person interview)	MA town/city

Interview candidates were contacted by email and received a recruitment message and one-page factsheet on the interview process. The recruitment communication indicated that the study needed interviewees who were willing to share their experiences and opinions on one or more of the following areas: i) various ways sharps enter and exit the home; ii) stakeholders who are directly or indirectly involved in sharps device procurement, use, and disposal in the home; and iii) policies, programs, or other initiatives that have either helped or hindered the routine use of sharps with injury prevention features. Expertise criteria for interview inclusion were as follows: nurses and other practicing clinicians at leadership or supervisory positions in HHC agencies; infection preventionists and occupational safety and health (OSH) professionals who can offer insights on BBP exposure prevention in HHC; or other specialists who could provide valuable insights on manufacturing, use, procurement, and/or disposal of sharps in HHC. Four large HHC agencies in Massachusetts were targeted for interview recruitment and three agreed to participate. At the planning stage, it was estimated that 20 interview sessions comprising different stakeholders could lead to theoretical saturation and this was suitable within the initial budgeting and time planning boundaries. Later on, the content analysis and suggestions from interviewees determined that it was beneficial to add more interview participants (e.g. diabetes educators, public health officers and health agents, other HHC agency representatives, and sharps’ manufacturers) than initially planned to provide multiple perspectives for the overall study findings.

### Data collection

Interview scripts and protocols were approved by the University of Massachusetts Lowell’s Institutional Review Board. Table 2 shows the major themes for which information was gathered via the interview scripts. The interviews were no longer than 60 minutes in duration. HHC agencies allowed interviews during work hours and were compensated $40 per interviewee. Other interviewees were not compensated if the interview was during their own work time and was consistent with normal job responsibilities. Two research team members conducted interviews in English. Each interview participant provided signed informed consent.

**Table 2:** **The main themes of Safe Home Care sharps study for which qualitative data was collected via the interview scripts**

**The first level of interview themes coded**	
●	Participant occupation or expertise
●	Recent or important events related to sharps injuries, BBP exposures or preventive interventions
●	Sharps injury prevention developments in HHC or general
●	Sharps flow into the home
●	Procurement of sharps by HHC agency
●	Sharps exit homes – sharps disposal practices
●	Insurance carriers coverage for sharps
●	Physician’s influence on sharps choice
●	Pharmacy’s role in sharps safety
●	Re-use of sharps
●	Participant advice to various stakeholders and needs assessment on improving sharps safety
●	Other information (subcoding for such sub-themes as sharps devices and technology, agencies/organizations mentioned, medications requiring sharps use, specific case descriptions, missed information added by participant)

### Data analyses

All 26 interview sessions were audio-recorded and transcribed. Participants received their typed transcript and had the opportunity to correct or clarify their responses. All 26 typed transcripts were coded paragraph-by-paragraph with NVIVO Qualitative Research Software (version 9.2) to obtain 3- to 4-level coding of themes resulting in total almost 800 theme nodes. NVIVO allowed weighing of themes depending on the number of different interview sessions in which they were coded. A qualitative research investigator with OSH expertise and a nurse investigator coded the interviews. The final coding structure comprised 12 first level parent theme nodes; many parent themes were decided a priori based on the questions asked in the scripts (Table 2). Most subthemes under the parent nodes were emergent depending on the interview transcript content. Interview findings were triangulated between same and different stakeholders. Additional triangulation was performed between the findings of the present study and another study which the research team was conducting simultaneously to investigate a wide range of OSH hazards among home care aides [10]. The OSH study included seven HHC agencies different from those in this study and also gathered information on sharps injuries and other blood exposures.

An archival study was conducted to collect and review both publicly available materials and internal documents pertaining to sharps procurement, reimbursement, use, and disposal in the HHC setting. Archival materials included book chapters, articles, training materials, HHC agency policies, photographs, and email correspondence. These were also coded thematically using NVIVO software.

Interview and archival study themes were integrated and summarized in the form of maps to illustrate how sharps enter and exit a home and the location of key decision-makers relative to other stakeholders. The integrated information then was analyzed using the “Hierarchy of Controls,” to assess the key points in the sharps systems that could be leveraged for effective interventions. The Hierarchy of Controls is an established framework to identify effective OSH/ public health interventions [15]; it holds that the most effective intervention is complete elimination of the hazard. The second most effective strategy is substitution, followed by engineering controls and administrative controls. Personal protective equipment (PPE) is considered the least robust hazard control method, however, sometimes PPE is the only available method of protection (e.g., in healthcare).

## Results

### Flow of sharps into the home

**Figure 1:**
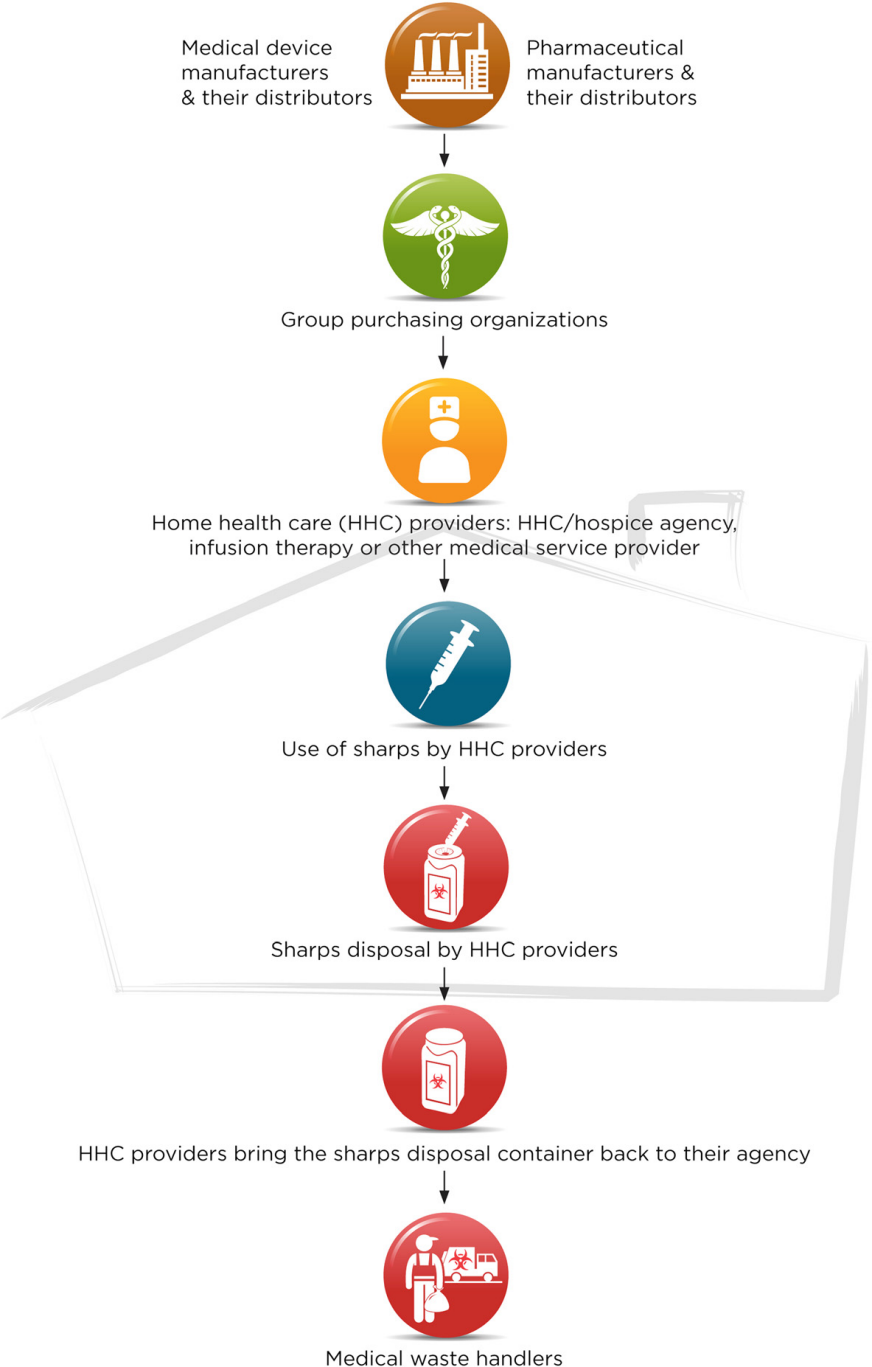
Flow of sharps into the home via HHC agency, hospice or other medical service provider. The background shadowing in the form of a house indicates which steps and stakeholders are located inside and outside the home environment. The HHC sharps system is much more extensive than the home itself which contains only a portion of the system.

**Figure 2:**
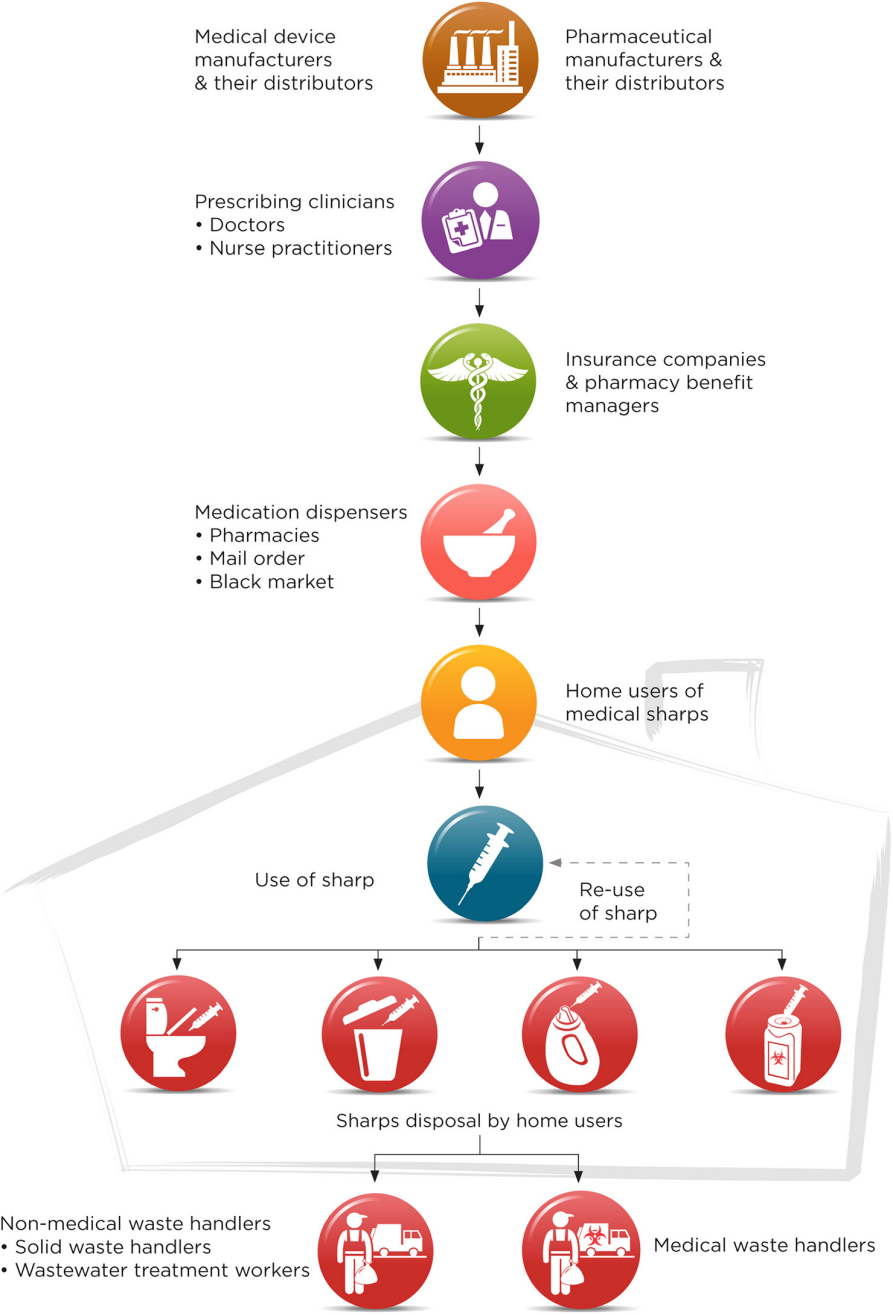
Flow of sharps into and out of the home for home users. The background shadowing in the form of a house indicates which steps and stakeholders are located inside and outside the home environment. The HHC sharps system is much more extensive than the home itself which contains only a portion of the system.

Figure 1 shows the flow of sharps into the home via the different types of HHC providers; Figure 2 shows the more complex situation of sharps flow into and of the home for home users. In both figures, the background shading in the form of a house indicates that only a portion of the sharps system is contained in the home. Most of the stakeholders and their contributions occur outside of the home environment.

A HHC patient may receive sharps from both situations simultaneously or sequentially. Sharps may enter the home via agency or hospice clinicians (e.g., visiting nurse or nurse practitioner), other medical service providers (e.g., infusion therapy company) (Figure 1), or a patient or family member may purchase sharps to address the patient’s needs (Figure 2). The route depends on the patient’s medical treatment. For example, a visiting clinician may provide sharps for an IV procedure. Alternatively, if a hospice patient needs a subcutaneous pump for pain management or medication, equipment may be ordered through a hospice pharmacy and shipped to the home. Interviewees noted that agencies and hospices are increasingly subcontracting IV therapies to infusion companies or other medical service providers.

Many patients self-administer injections for medical treatments, such as for diabetes, multiple-sclerosis, and blood clots. In these cases, patients or family members typically buy prescribed medications and needed sharps at pharmacies, specialty pharmacies, mail-order, or from an unauthorized “black-market” source, including the internet. Some medications are supplied in prefilled syringes (e.g., insulin or blood thinners) or in kits containing sharps and other supplies.

### Use of sharps in the home

The HHC providers who were interviewed in this study, reported that their agency clinicians used sharps with injury prevention features (SIPFs). Agency representatives shared challenges experienced by their clinicians using SIPFs, in particular activating them; e.g., the sliding sheath on a syringe did not properly engage and unexpectedly slid back exposing the needle, or retractable syringes did not retract because the plunger was not fully depressed.

Patient-procured devices generally do not have SIPFs. Most interviewees reported that re-use of sharps by home users for cost savings and convenience is common (Figure 2). Re-use presents an occupational hazard for clinicians and aides: unsecured sharps may be encountered unexpectedly (e.g., in trash, bedding, chair cushions, on tables). A sharps manufacturer cited testimonies from patients re-using insulin syringes up to 20 times. A sharps safe disposal advocate described re-use among home users (interview session #11, Table 1):

*Absolutely, oh yes, sadly they do. . . to save money. . . I mean, if you’re a diabetic and you’re on a budget and you have to give yourself three or four shots a day, they’ll use that same needle, most of these people don’t see how that needle deteriorates every time you use it, and don’t understand the chance of infection . . . diabetes is an expensive disease.*

A HHC agency educator explained the problem with patients’ reusable lancet pens (session #7):

*And then something around these lancet pens. . . it looks like an insulin pen, but it’s triggered with a spring loaded device. And they’re nasty. Because you’ve got to take the cap off. And then literally with your fingers, you have to go in and pull out this lancet. I mean, you could do it with tweezers. You could do it with forceps if you had them. And it’s tiny. . . But patients use [lancet pens] over and over and over again. . . they save on the cost of the lancets.*

A HHC agency infection preventionist described a patient and family member sharing a lancet -- this lancet had recently caused a sharp injury to an agency employee (session #1): “*. . . the sharps injury happened yesterday, it was in a different office. . . I think the patient and the son are both using the [same] lancet“.*

### Flow of sharps from the home

HHC clinicians and service providers (e.g., infusion treatment) normally carry disposal containers for their sharps, which are returned to their organization for pickup by a hazardous waste management company (Figure 1). If clinicians use a patient’s sharp device, it typically goes into the patient’s sharps disposal container. The HHC agency infection preventionist described the significance of educating staff on using disposal containers correctly. The agency’s containers were leak-proof when tightly capped but some clinicians just placed the cap loosely, allowing fluid leakage. She explained the importance of placing the container upright in the clinician’s bag (interview session #1): *We have a side pouch that hangs off the clinician’s bag and it zips and that keeps it upright and that's the point of the sharps container. . . it has to be upright.*

Options for patients or family to safely discard medical sharps include community waste collection sites, sharps return-by-mail boxes, or disposal kiosks. On rare occasions, a pharmaceutical company provides a sharps disposal container with their medication. Clinicians teach patients to use a rigid puncture-resistant, opaque container (e.g., laundry detergent bottle) for collecting and disposing sharps. Patients are instructed to fill the container not more than three-quarters full, cap it tightly and tape the cap before throwing it into household waste. Throughout the United States, many home users and family members still throw used sharps into the trash. Elderly residents have been seen bringing used sharps in plastic bags to municipal waste collection sites. Three interview sessions reported home users flushing syringes down the toilet. A local public health officer shared (session #21):

*They have people flushing these down the toilet, and they were ending up on the racks of the sewer plant. So Wastewater Plant is willing to provide collection to try to keep them out of the sewer system because it’s an occupational hazard to their employees, because they have bar racks that pick up things like hypodermic needles like this and they have to actually break them off and put them in bags . . .wastewater workers are trying to keep the hypodermics out of the sewer.*

Figure 2 illustrates home users’ sharps disposal practices. To prevent sharps injuries, most interviewees emphasized the importance of safe, convenient, and no-cost sharps disposal options for home users. A sharps manufacturer expressed the following when asked for advice on sharps injury prevention (session #14): “*. . .the number one thing would be free sharps containers. . .and number two would be a convenient drop-off and pick-up for these sharps containers”.* A drop-off station in every town was considered important; a diabetes educator expressed empathy for a patient whose small town had no sharps collection and who was unable to drive to the nearest drop-off site.

### Location of the most effective intervention points in the sharps HHC systems map

Table 3 summarizes the priorities for public health interventions to prevent sharps injuries obtained by assessing the systems maps and supporting data using the OSH Hierarchy of Controls. Column 1 in Table 3 shows the intervention levels in the hierarchy and column 2 shows the location of the intervention level on the sharps systems maps (Figures 1 and 2). Column 3 gives specific examples of interventions that were identified from the interview or archival data analysis, while column 4 gives the frequency with which an intervention was cited in the interviews and column 5 gives literature citations for the interventions. The best way to improve overall sharps safety in HHC is to eliminate the sharp, for example by substituting it with an alternative treatment that avoids the sharp entirely (elimination/substitution, Table 3). This removes the sharp hazard completely and impacts the largest number of people in the system. If a sharp procedure cannot be eliminated, an effective primary prevention method is use of SIPFs (engineering controls, Table 3). The study findings prioritize interventions with stakeholders at the beginning of the systems map where the potential for sharps injuries across the entire HHC system can either be eliminated completely or at least substantially reduced with SIPFs.

**Table 3:** **Sharps injury prevention in home healthcare according to Hierarchy of Controls**

**Sharps injury prevention/control method**	**Systems maps location of stakeholders to intervene (see Figures** 1 **,** 2 **)**	**Intervention examples cited both in the study interviews and literature**	**Study interviews: citation frequency on interventions**	**Literature evidence on interventions**
Elimination/ substitution	Beginning	● Eliminate unnecessary injections/unnecessary sharps	High (60% or more)	[16-22,27,40,41]
		- e.g. needleless IV-systems		
		● Apply needleless medication alternatives		
		- e.g. jet injectors, aerosols via inhalation, mucosal vaccines tablets, transdermal patches		
Engineering controls	Beginning and middle	● Design and use sharps with injury prevention features	High (60% or more)	[11,16,26,27,40,41]
		- e.g. existing retracting, sheathing, blunting technologies		
		- e.g. new sharps innovations		
		● Design and use sharps disposal containers		
Administrative controls	Middle	● Promote and educate on safe use of sharps devices	High (60% or more)	[16,40-42]
		● Promote and educate on safe sharps disposal container use and community disposal practices		
		● Implement and annually review a BBP exposure control plan		
		● Ensure work practices in line with an exposure control plan		
		● Minimize re-use among home users when possible		
Personal protective equipment	Middle and end	● Use gloves/double-glove	Moderate (less than 50%)	[16,26,40-42]
		●Use puncture resistant gloves		
		● Apply protective clothing		
		- Goggles, face shields, masks, gowns		
		- Other barriers/ filters		

Sharps injury prevention methods, stakeholders’ location in the systems maps who can act at each method level, specific intervention examples, citation frequency in study interviews, and examples of literature documentation.

Sharps manufacturers and designers are best positioned to develop needleless instruments or SIPFs. Many interviewees praised SIPFs. A HHC agency representative brought up that better standardization among SIPFs would streamline training efforts and make sharps use more consistent and intuitive as sharps from different manufacturers function differently, each type requiring a learning curve and focused attention when switching from one to another. Other sharps safety stakeholders include pharmaceutical companies, group purchasing organizations, physicians and other prescribing clinicians, pharmacies/pharmacists, and insurance providers; of these, pharmaceutical companies and health insurance providers have the greatest injury prevention leverage. Drug manufacturers are positioned to develop medical treatments delivered without needles. Health insurers control access to SIPFs for home users through their approved products and reimbursement policies.

The study interviewees advocated for robust sharps disposal containers (an engineering control). Stakeholders both at the beginning and middle of the maps have the greatest leverage for injury prevention through improving disposal approaches (Figures 1 and 2). Among administrative controls, the roles of education and advocacy were dominant patterns in particular both in sharps device and disposal container use as well as instituting safe work or care practices. Personal protective equipment (PPE) was cited least frequently as an avenue for sharps injury prevention. Stakeholders at the end of the maps have the highest injury risk resulting from poor disposal practices and they can rely mostly on administrative controls and PPE only (Figures 1 and 2).

## Discussion

This study has highlighted the importance of interventions at the beginning of the HHC sharps system through eliminating/substituting sharps or with engineering controls such as SIPFs and robust disposal containers. However, the study also supports that engineering controls must go hand-in-hand with education and standard precautions principles for effective protection from BBP exposures. When triangulating findings between different studies, the research team’s large OSH home care aide study emphasized the importance of teaching safety at new employee orientation -- one supervisor of a large HHC agency stated that their in-depth orientation training with an intensive BBP exposure prevention component was viewed as a major factor in curtailing the agency’s sharps injuries [10]. Literature also confirms the combination of preventive approaches for the best results; for example U.S. Centers for Disease Control and Prevention’s (CDC) *Workbook for Designing, Implementing and Evaluating a Sharps Injury Prevention Program* advocates multi-component prevention approaches whereby elimination/substitution and engineering controls are supported by education and formation of needlestick prevention work groups in HHC agencies [16].

### Preventing sharps injuries at the beginning of the systems: needleless treatment methods

Although eliminating and substituting sharps may sound challenging, needleless treatment methods – such as jet injectors, inhaled aerosols, and other needle-free methods -- are already on the market. A jet injector can deliver, via high pressure, a prescribed drug, vaccine, or other medication intra-dermally, subcutaneously, or intramuscularly [17]. In addition to eliminating sharps injuries and hazardous BBP waste, the literature has reported other jet injector advantages. These include increased capacity for rapid immunization of large populations, less technique sensitivity for achieving different subcutaneous penetration depths, elimination of plunger aspirations, and improved tissue dispersions [17,18]. A clinical trial study on jet injection of poliovaccine delivery among 400 infants in Oman also measured parents’ preference on the delivery method for the next vaccination. Among 185 parents who responded 8 (4%) preferred needle-syringe vaccines, 172 (93%), preferred a needle-free method, and 5 (3%) had no preference [19]. The disadvantage is cost: a 2013 source reported a base price for a conventional needle injection about 0.05 U.S. dollars versus 1 U.S. dollar for a needleless jet injection [17]. However, this estimate excludes downstream costs: the total costs of an injection include sharps waste and disposal costs as well. It is also important to remember that sharps injuries are costly and needle-free device prices decrease with greater demands. Mucosal vaccinations, in particularly via oral and nasal routes, have several advantages: easy to administer, natural route for mucosal and systemic response, and possible cost-savings due to lower dose needs for live vaccines [18]. Furthermore, aerosol therapies have developed over the years and such treatment examples now comprise drug deliveries by inhalation (e.g. oral inhalation of insulin), inhaled gene therapy (e.g. treating cystic fibrosis), and vaccination by inhalation (e.g. inhaled measles or flu vaccine) [20-23]. Future studies will examine the success of these newer therapies.

### Importance of SIPFs and impact of public policy

If a sharp cannot be substituted with a needleless treatment method, SIPFs are an essential primary prevention measure against percutaneous injuries. As aforementioned, HHC agencies are required by the OSHA’s Bloodborne Pathogens Standard use engineering and work practice controls to eliminate or minimize BBP exposures. SIPFs are an essential component of the engineering controls. Despite this OSHA’s requirement, the earlier study found that SIPFs were not frequently used [5].

The study findings emphasize that the most effective sharps injury prevention exists at the beginning of the systems map (Figures 1–2, Table 3), where both sharps device and pharmaceutical manufacturers can eliminate or drastically reduce sharps injury potential with needleless systems and SIPFs. Furthermore, both device manufacturers and insurance providers can improve safety with more affordable and accessible SIPFs for home users. Physicians typically specify sharps only in terms of function for the prescribed medication, such as an insulin syringe that makes the dose easy to read and measure accurately. None of interviewees knew of a physician specifying “safety device”; rather the prescription would indicate, for example, “*25-gauge subcutaneous 3 cc syringe, or an insulin syringe*.” Nonetheless, physicians are important advocates to influence sharps manufacturers and insurance companies for wider use of SIPFs among home injectors. Physicians can also educate patients on availability of SIPFs. The same pertains to pharmacies.

The Needlestick Safety and Prevention Act (NSPA) of 2000 that revised the OSHA’s BBP Standard has improved sharps safety in hospital settings. In Massachusetts, a study analyzed trends in sharps injury rates among employees of 76 acute care hospitals which had reported 16,158 sharps injuries to the state surveillance system during 2002 – 2007: within this time period, the annual sharps injury rate had decreased by 22% and the injury rates involving devices for which SIPFs were available and increasingly used had declined [24]. Another study of sharps injury data from 85 hospitals estimated that more than 100,000 sharps injuries were prevented every year during 2001–2005 with a cost-saving of $69-415 million [12]. Nonetheless, the NSPA impact is unclear for non-hospital healthcare settings. The 2012 Consensus Statement and Call to Action by U.S. organizations involved in sharps injury prevention acknowledged that use of SIPFs in non-hospital health care settings – i.e. HHC, long-term care, practitioners offices and clinics -- had been less consistent [25]. The Statement addressed a critical need for obtaining valid and reliable sharps injury data targeting non-hospitals. One of the recommendations called for support from government agencies, such as OSHA, to promote regional emphasis programs on enforcement of the BBP standard and CDC to support epidemiological research for non-hospital settings. Accrediting and licensing organizations (e.g., the Joint Commission) and workers’ compensation insurers can influence healthcare worker safety by enhancing compliance incentives. Furthermore, professional organizations and product distributors can facilitate the flow of appropriate devices and educational materials for non-hospital settings [25]. Among others, the International Sharps Injury Prevention Society provide comprehensive lists of SIPSs as well as other products and tools to improve BBP exposure prevention [26]. Recently, the World Health Organization (WHO) released a global guideline on the use of safety-engineered syringes for injections in healthcare settings [27].

### Re-use of sharps

Re-use of medical sharps is widespread among home users; the main reason is to save money. Convenience is also a factor for patients requiring several injections a day, and re-use may satisfy a desire to generate as little non-biodegradable waste as possible [28]. Furthermore, there are not good sharps disposal options available in public and users do not want to carry many needles along. Little is known about health risks related to sharps’ re-use; reports of adverse health outcomes are rare. One research team studied lipohypertrophy -- “*a thickened ‘rubbery’ swelling of tissue*” -- among 430 outpatients injecting insulin; one risk factor was sharps re-use, with risk increasing significantly when sharps were used more than 5 times [29]. Some sources suggest there can be needle tip damage due to re-use, however, one study concluded that using insulin pens four to five times a day does not affect the needle tip or increase pain intensity [30]. It has also been reported that the most important driver for medical product selection is whether it is reimbursed by the country’s health service system or insurance; for example, U.S. citizens and residents relying on Medicaid have the fewest choices in medical technology [31]. Sharing of insulin pens is also a concern. The U.S. CDC has issued an important clinical reminder not to use multi-dose insulin pens for more than one person even when the needle is changed because after injection blood can flash back into the insulin cartridge, thus, creating a bloodborne pathogen transmission risk if the pen is used by more than one person [32].

### Sharps disposal in communities

The study interviews showed that interventions to encourage safe sharps disposal policies and practices in communities remain a priority. The interviewees highlighted that sharps disposed of unsafely in communities are a serious occupational risk to waste management handlers. Sharps are even flushed through the toilet. In addition to this study, a survey conducted in New Jersey among 44 diabetic patients, 86% of respondents reported improper disposal -- 7% flushed sharps through the toilet [33].

There are no federal regulations or consensus standards that direct consumers on how and where to dispose of sharps; any disposal regulations are at the county or local level [34]. The Environmental Protection Agency’s guidelines on disposal of medical sharps are too general for consumers [35]. Similarly, no approved consumer guidelines exist on how to choose a sharps disposal container, although increasingly laundry detergent bottles have been recommended if Food and Drug Administration (FDA)-approved sharps containers are not available [34]. In 1998, the National Institute for Occupational Safety and Health published guidelines on selecting sharps containers for the workplace [36]. These guidelines provide comprehensive directions on container selection, evaluation, and use; however, they are rather long and complex for consumers. A similar, consumer-friendly tool could be developed that would guide consumers on selection and proper use of sharps containers. One of the interviewees (a sharps disposal advocate) brought up the FDA’s role in incorporating increased sharps disposal guidance into the existing drug labeling requirements (interview session #12):

*You know, the FDA has to clear these drugs with something called a 510 K pre-market notification process. But why can’t they also require, alert the drugging effort to provide a list of the community drop-off centers or kickback programs or hey, call 1–800, US-FDA and we’ll tell you based on your zip code where your local drop-off point is.*

At least two states – California (2008) and Massachusetts (2012) – have banned sharps in household waste and require special disposal [37,38]. As of July 2014, the Massachusetts Department of Public Health listed 295 sharps disposal sites for Massachusetts and some of them were funded by the Department of Public Health [39]. In reality, disposal options are highly variable from one city or town to another. Some pharmacies, hospitals, clinics, and HIV programs may accept used needles and syringes. A representative of a sharps manufacturer explained that the majority of corporate U.S. pharmacies are not very supportive of their individual pharmacies taking back used sharps because of concerns about injury and possibility of litigation. However, programs have been established—often in cooperation with a municipality—for pharmacies to voluntarily take sharps back. Another sharps manufacturer suggested that pharmacies could become less resistant if a sharps disposal program was based on a positive reward system. A similar reward system principle could work for home users (session #16): “*You bring them back and we’ll reward you with free needles or we’ll reward you with a point system that gives them something in return for good compliance*.”

## Conclusions

This study developed two maps of the flow of sharps into and out of home and the relative location of stakeholders. The most effective intervention opportunities are at the beginning of the map, where hazard elimination could be targeted: i) drug manufacturers by developing medical treatments with needleless methods; ii) sharps manufactures by designing affordable needleless instruments or SIPFs; and iii) health insurance providers by providing devices affordably for home users through their product listing and reimbursement policies. A particular challenge of intervening with these stakeholders is that they are not at risk of sharps injuries and are not directly impacted by the occurrence of sharps injuries.

The study findings show that the sharps system in HHC is complex and that only a portion of the system is actually located in the home environment. Therefore, comprehensive public health interventions need to extend throughout industry and the community. Interventions focused solely on the home environment will not offer long-term effectiveness because they do not address the root cause – i.e. the presence of a contaminated sharp. Nonetheless, interventions are needed in the home setting for safe sharps disposal practices and to minimize re-use among home-users whenever possible. Training on sharps injury prevention is needed for all stakeholders in systems of sharps use in HHC. The figures and table developed here are intended to assist with training development.
